# Latent tuberculosis infection treatment completion in Biscay: differences between regimens and monitoring approaches

**DOI:** 10.3389/fmed.2023.1265057

**Published:** 2023-10-31

**Authors:** N. Ortiz Laza, I. Lopez Aranaga, J. Toral Andres, B. Toja Uriarte, B. Santos Zorrozua, L. Altube Urrengoechea, J. Garros Garay, E. Tabernero Huguet

**Affiliations:** ^1^Pulmonology Service, Cruces University Hospital, Biocruces-Bizkaia Health Research Institute, Barakaldo, Spain; ^2^Pulmonology Service, Galdakao-Usansolo Hospital, Galdakao, Spain; ^3^Pulmonology Service, Santa Marina Hospital, Bilbao, Spain; ^4^Biocruces Bizkaia, Barakaldo, Spain

**Keywords:** latent tuberculosis infection, treatment, adherence, nurse case manager, telemonitoring

## Abstract

**Introduction:**

Contact tracing and treatment of latent tuberculosis infection (LTBI) is a key element of tuberculosis (TB) control in low TB incidence countries. A TB control and prevention program has been active in the Basque Country since 2003, including the development of the nurse case manager role and a unified electronic record. Three World Health Organization-approved LTBI regimens have been used: isoniazid for 6 months (6H), rifampicin for 4 months (4R), and isoniazid and rifampicin for 3 months (3HR). Centralized follow-up by a TB nurse case manager started in January 2016, with regular telephone follow-up, telemonitoring of blood test results, and monitoring of adherence by electronic review of drugs dispensed in pharmacies.

**Objective:**

To estimate LTBI treatment completion and toxicity of different preventive treatment regimens in a real-world setting. Secondary objective: to investigate the adherence to different approaches to preventive treatment monitoring.

**Methods:**

A multicentre retrospective cohort study was conducted using data collected prospectively on contacts of patients with TB in five hospitals in Biscay from 2003 to 2022.

**Results:**

A total of 3,066 contacts with LTBI were included. The overall completion rate was 66.8%; 86.5% of patients on 3HR (*n* = 699) completed treatment vs. 68.3% (*n* = 1,260) of those on 6H (*p* < 0.0001). The rate of toxicity was 3.8%, without significant differences between the regimens. A total of 394 contacts were monitored by a TB nurse case manager. In these patients, the completion rate was 85% vs. 67% in those under standard care (*p* < 0.001). A multivariate logistic regression model identified three independent factors associated with treatment completion: being female, the 3HR regimen, and nurse telemonitoring.

**Conclusion:**

3HR was well tolerated and associated with a higher rate of treatment completion. Patients with nurse telemonitoring follow-up had better completion rates.

## Introduction

Contact tracing and treatment of latent tuberculosis infection (LTBI) are key elements of the “prevention package” of the END TB strategy. LTBI is defined as a state of persistent immune response to stimulation by *Mycobacterium tuberculosis* antigens with no evidence of clinically manifest active tuberculosis (TB). LTBI treatment is particularly important in countries with a lower tuberculosis incidence, where a higher proportion of cases are due to reactivation of latent infection (1).

The Department of Health of the Basque Country, which in 2002 had a rate of 26.2 cases per 100,000 inhabitants, launched a TB control and prevention program which has been active since 2003. This program includes a TB nurse case manager in each area and a specific unified electronic record that stores data on both patients diagnosed with tuberculosis and the corresponding contact tracing. At present, the TB rate has dropped to 8.6/100,000 inhabitants in Biscay (2, 3).

Technological advances and the growing evidence of the importance of monitoring adherence to LTBI treatment led to the launch in January 2016 of centralized monitoring of the LTBI treatment by a TB nurse case manager. This was an innovation compared to the traditional standard follow-up in regular scheduled medical consultations with each specialist in the corresponding area.

Currently, three WHO-approved LTBI regimens are available in our country for the treatment of LTBI: 6 months of isoniazid (6H), 4 months of rifampicin (4R), and 3 months of isoniazid and rifampicin (3HR). These have similar efficacy and adverse effects, but shorter treatments have shown better adherence, however, there are few studies in our setting (3–6).

We undertook this study to assess the LTBI treatment completion rate in contacts in whom such treatment was recommended in our area. As secondary objectives, we aimed to investigate the results of treatment according to the different follow-up approaches and regimens, as well as to describe toxicities associated with the different preventive treatments and to analyze other factors associated with non-adherence.

## Methods

This was a multicenter retrospective cohort study that used prospectively collected data on contacts of smear-positive TB patients from five hospitals in Biscay from 2003 to 2022, Santa Marina University Hospital, Galdakao University Hospital, Cruces University Hospital, San Eloy Hospital and Urduliz Hospital. Pseudo-anonymized data were extracted from the specific electronic program for regional TB control.

### Study population

All contacts of people with smear-positive pulmonary or laryngeal TB in Biscay from 2003 to 2022 recorded in the electronic database of the regional TB control program. Patients diagnosed with active TB during the contact study were excluded from the analysis.

### Variables

The primary variable was LTBI treatment outcome: completed [defined as taking >80% of doses of the prescribed medication regardless of treatment changes, within 12 and 18 months from the start for rifampicin-containing and isoniazid-alone therapies, respectively (7)], dropped, withdrawn due to intolerance, refused, and lost to follow-up.

Exposure variables: standard monitoring versus tele-follow-up by a nurse case manager, type of regimen: 6H, 4R, or 3HR. Other variables analyzed included age, sex, date, nationality (grouped by WHO region), degree of contact and toxicity (1).

The TB nurse case manager collected data on index cases and their contacts. Patients were studied according to the local guidelines, using a system of concentric circles representing the amount of time spent with the index case, classifying contacts as intimate (daily and more than 6 h, mostly households), regular (daily and less than 6 h) or sporadic (not daily). The latter were studied in outbreaks or if the index TB case was high risk (extensive tuberculosis, long diagnostic delay and high bacillary load).

For the diagnosis of LTBI, TST (tuberculin skin test) was used until 2012 and since then, a 2-step strategy with TST using QuantiFERON as confirmatory test similar to that described by Muñoz et al. (8).

During the first years of the study period, in all areas, these contacts were referred to the clinic where the index case was diagnosed or to their primary care center. As of January 2016, three hospitals started centralized monitoring of LTBI treatment by the corresponding TB nurse case manager, following assessment and prescription of LTBI by a pulmonologist. The nurse case manager carried out regular telephone follow-up, telemonitoring of scheduled blood test results, and monitoring of adherence through electronic review of the medication dispensed in pharmacies, as well as being available via direct channels of communication (mobile phone and WhatsApp) to address patients’ concerns and monitor possible side effects. The monitoring of LTBI treatment in the other hospitals continued in the traditional way.

The study was approved by the regional ethics committees in accordance with the Declaration of Helsinski’s guidelines for research in humans. Patient privacy and the confidentiality of personal data have been safeguarded in line with the provisions of European law (9).

## Statistical analysis

Continuous variables are reported as the mean (standard deviation) for normally distributed data and otherwise as the median (interquartile range). Categorical variables are presented as frequency (percentage). When comparisons were made between two groups, Student’s t-test was used, in the case of normally-distributed continuous variables, and otherwise the Kruskall-Wallis test. Chi-squared or Fisher’s exact tests were used for the categorical data. To construct the multivariate logistic regression model, the dependent variable was the final LTBI outcome, and the independent variables were age, sex, date, degree of contact, type of follow-up, and type of regimen. Multivariate analysis was performed using a multivariate logistic regression model, including variables with *p*-values lower than 0.100 in the bivariate analysis as predictors. We eliminated the variables with the highest *p*-values one at a time until all the variables entered were significant (*p*-value <0.05). The Hosmer-Lemeshow goodness-of-fit test for logistic regression was used to assess the model fit. Differences were considered statistically significant when *p* < 0.05. All analyses were performed using R statistical software (version 4.0.1 R: A language and environment for statistical computing. R Foundation for Statistical Computing, Vienna, Austria).

## Results

**Figure 1:**
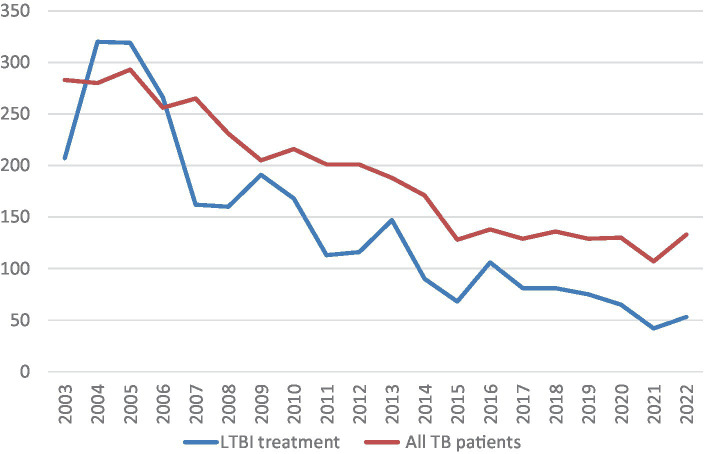
Evolution of TB cases and LTBI treatments 2003–2022.

A total of 17,817 persons were studied from January 2003 to December 2022 in Biscay, corresponding to 3,820 TB patients. Only 2,267 (59.3%) of them were smear or culture positive pulmonary or laryngeal TB and had contact tracing (7,8 persons per contact study on average). A contact study was also performed in children and pleural TB to try to identify the index case. Among the total sample, 132 (0.74%) new cases of TB disease were diagnosed, and 3,066 (17,2%) contacts were diagnosed with LTBI with an indication for treatment. During this long period of time the incidence of tuberculosis has decreased in our area and in parallel the total number of contacts studied, as shown in Figure 1.

The median age of contacts with an indication for LTBI treatment was 32.5 years (21.1–44.2) and 46.6% were women. The most frequent place of origin after Spain (74,8%) was the Region of the Americas (11.4%) followed by the Eastern Mediterranean Region (5.6%) (Table 1).

**Table 1:** Baseline characteristics stratified by adherence to latent tuberculosis infection treatment outcome.

	All	Completed	Not completed	OR	Ratio *p* value	Overall *p* value
	*N* = 2,962	*N* = 2048	*N* = 914			
**Age**, median [25th;75th]	32.3 [20.9;44.1]	32.6 [20.2;44.9]	31.7 [21.8;41.8]	1.00 [0.99;1.00]	0.198	0.365
**Sex, *N* (%)**						0.002
Male	1,588 (53.6%)	1,059 (66.7%)	529 (33.3%)	Ref.	Ref.	
Female	1,374 (46.4%)	989 (72.0%)	385 (28.0%)	0.78 [0.67;0.91]	0.002	
**Nationality, *N* (%)**						<0.001
Spain	2,216 (74.8%)	1,576 (71.1%)	640 (28.9%)	Ref.	Ref.	
Americas	340 (11.5%)	216 (63.5%)	124 (36.5%)	1.41 [1.11;1.79]	0.01	
Eastern Mediterranean	166 (5.60%)	114 (68.7%)	52 (31.3%)	1.12 [0.79;1.57]	0.50	
Europa	117 (3.95%)	73 (62.4%)	44 (37.6%)	1.49 [1.00;2.18]	0.04	
Africa	113 (3.81%)	63 (55.8%)	50 (44.2%)	1.95 [1.33;2.86]	0.01	
Western pacific – Asia	10 (0.34%)	6 (60.0%)	4 (40.0%)	1.66 [0.41;6.01]	0.45	
**Follow-up, *N* (%)**						<0.001
Traditional follow-up	2,535 (86.5%)	1712 (67.5%)	823 (32.5%)	Ref.	Ref.	
Nurse telemonitoring follow-up	394 (13.5%)	336 (85.3%)	58 (14.7%)	0.36 [0.27;0.48]	<0.001	
**Regimen, *N* (%)**						<0.001
6H	1844 (67.6%)	1,260 (68.3%)	584 (31.7%)	Ref.	Ref.	
3HR	808 (29.6%)	699 (86.5%)	109 (13.5%)	0.34 [0.27;0.42]	0.00	
4R	76 (2.79%)	57 (75.0%)	19 (25.0%)	0.72 [0.41;1.21]	0.22	
**Year, *N* (%)**						0.017
2003– > 2007	1,222 (43.9%)	911 (74.5%)	311 (25.5%)	Ref.	Ref.	
2008– > 2012	746 (26.8%)	517 (69.3%)	229 (30.7%)	1.30 [1.06;1.59]	0.01	
2013– > 2017	488 (17.5%)	343 (70.3%)	145 (29.7%)	1.24 [0.98;1.56]	0.07	
2018– > 2022	327 (11.7%)	250 (76.5%)	77 (23.5%)	0.90 [0.68;1.20]	0.48	

Ref: reference.

Nearly half (49.5%) were household contacts, and there were no differences regarding treatment completion rate according to the degree of contact.

Overall, treatment was completed by 66.8% of the contacts with a treatment indication. A total of 115 (3.8%) of the contacts withdrew due to intolerance, with no significant differences in toxicity between the different regimens: 26 with 3HR (3.2%), 3 with 4R (3.9%), and 85 with 6H (4.6%). Hepatoxicity was the most common adverse event (91% of them). Just 6.1% dropped out of treatment, while 174 contacts (5.7%) refused treatment. Further, 304 (9.9%) of the contacts with an indication for LTBI were lost to follow-up and 104 had missing data concerning treatment outcome (the latter being excluded from the analysis).

Follow-up involved telemonitoring by the nurse case manager in 13.3% of contacts, while 86% received traditional follow-up by the corresponding pulmonology or primary care clinic. As for the regimens, only those accepted by the WHO and available in our country were taken into account for the analysis. The most frequently recommended regimen was 6H (67.6%), followed by 3HR (29.6%). There were no differences in baseline demographic characteristics among patients with the different regimens.

According to final status, in bivariate analysis, completion rates were higher in women (72%, OR: 0.78; 95% CI: 0.67–0.91), those born in Spain (71%), those who received the 3HR regimen (86.5%, OR: 0.34; 95% CI: 0.27–0.42), the 4R regimen which is also a short regimen was not statistically better probably due to small number, and contacts telemonitored by a nurse case manager (85.2%, OR: 0.36; 95% CI: 0.27–0.48) (Table 1). In the multivariate analysis, female sex (OR = 1.26; 95% CI: 1.06–1.51), the shorter regimen 3HR (OR = 2.90; 95% CI: 2.29–3.70), and tele-follow-up by a nurse case manager (OR = 1.75; 95% CI: 1.27–2.45) remained significantly associated with completing treatment (Table 2). The Hosmer-Lemeshow goodness-of-fit test for logistic regression gave a *p* value of 0.852.

**Table 2:** Multivariate analysis of factors associated with completing latent tuberculosis infection treatment.

Baseline characteristic	AOR^1^	95% CI^1^	*p*-value
**Sex**			
Male	1.000	—	
Female	1.268	1.06, 1.51	0.009
**Regimen**			
6H	1.000	—	
3HR	2.902	2.29, 3.70	<0.001
4R	1.358	0.804 2.40	0.270
**Follow-up**			
Standard	1.000	—	
Nurse telemonitoring	1.757	1.279, 2.456	<0.001

^1^AOR, adjusted odds ratio; CI, confidence interval.

## Discussion

This multicenter study provides the results of real-world LTBI preventive treatment in contact studies over 20 years of the TB program in the province of Biscay.

In our study, only two-thirds (66.3%) of all patients with an indication for treatment completed the course prescribed, similar to rates found in Canada and the USA by Sullivan and Hirsch-Moverman (10, 11), and lower than those of 79% reported in Italy and 80%–89% in two multicenter studies in Spain (7, 12, 13). A systematic review of 83 studies on this topic described rates ranging from 46% to 96%, depending on the characteristics of the population studied, being much lower in homeless people than in healthcare workers (14).

Overall, 408 (13.3%) patients were lost to follow-up or had missing data on treatment outcome, comparable to results in research in North America (14.5%) (15). The rate of treatment adherence was lower among patients born outside Spain than among those born in this country in the bivariate analysis, but being foreign-born disappeared as a risk factor for non-completion of treatment in the multivariate analysis, and hence, we cannot draw definitive conclusions. The TITL treatment date also showed differences in the bivariate analysis but when included in the multivariate analysis the Hosmer and Lemeshow goodness of fit (GOF) test showed a very low value, probably because the COVID pandemic period altered the follow-up. The impact of COVID, while maintaining normal activity in our TB services, limited patient access to the healthcare system and led to a sharp decline in the number of TB cases and LTBI treatments in 2020 and 2021, as recently published in our country (16).

Analyzing our results by LTBI regimen, we found that the adherence rates are much higher with a shorter regimen, namely, 3HR, than the longer 6H, reaching as high as 86% vs. 68%. This has been widely described in previous studies, also in Spain by Jimenez-Fuentes, and is in line with current WHO and CDC recommendations (5, 6, 17–19). According to a meta-analysis some years ago, short regimens offer similar results in terms of protection with similar rates of adverse effects but much better adherence and lower costs (20). The use of these short regimens based on rifampicin is associated with 20%–40% higher adherence rates (21, 22) without significantly increasing the risk of rifamycin resistance (23). Further, there was no difference in toxicity between the different regimens, ranging from 3.2% with 3HR to 4.5% with 6H, similar to rates described in previous studies (18, 21).

Finally, one of the main findings of this study was that tele-follow-up by a nurse case manager significantly improved the rate of LTBI treatment completion, 85% versus 67% in the standard follow-up, with a statistically significant difference that was maintained in the multivariate analysis. This follow-up by a nurse with expertise in TB management allowed for comprehensive and individualized management (24). The tele-follow-up protocol included health education, and provision of oral and written information on latent TB infection and the indicated treatment and its possible side effects. The patients had easy access to a clinician with specialized training in TB for support, via direct channels of communication, for both addressing concerns and monitoring possible side effects, avoiding unnecessary travel for patients and reducing patient volume at healthcare centers. Some previous studies have highlighted that treatment non-completion was strongly associated not only with adverse effects but also with the inconvenience of clinic and pharmacy schedules (14, 17). On the other hand, technological developments have not only facilitated regular communication between patient and nurse case manager but also enabled close monitoring through the medical record and electronic review of the collection of medication from pharmacies. A recent review on the effects of digital health technologies in LTBI, suggests that they are at least equivalent to current practice (25). In addition, a study on immigrants in Israel showed that LTBI treatment supervised by an expert nurse with reduced physician follow-up was safe and proved to be cheaper than standard monitoring (9).

The main limitation of this study was the missing data. This was an analysis of data collected over 20 years, and many missing records could not be recovered. Nonetheless, since there was a large sample size, we believe that this will have had a minimal impact on the results. Another limitation is that confounding factors such as comorbidities, substance use, and socioeconomic status, identified in the literature as associated with low adherence, have not been analyzed (26). On the other hand, due to the long study period, the diagnostic methods have not been uniform, in particular, with very little use of QuantiFERON for the diagnosis of latent tuberculosis infection in the early years of the study. The same applies to the different treatment regimens, with the 3HR regimen only being widely used after 2012 and the 4R regimen after 2018. Lastly, we measured adherence based on self-report of missed doses and pharmacy records but did not collect empty boxes.

These results have clinical implication and show other aspects that could be improved in the management of TB contacts in our setting. In our study, non-Spaniards had worse treatment completion rates, so having cultural mediators and community health workers could help to improve completion rates as has been shown in asylum seekers in Sweden (27).

The use of rifapentine which is not available in our country at present, has shown higher treatment completion rates and could simplify treatment adherence (25, 28, 29).

In conclusion, to move towards TB elimination in low-incidence countries, efforts should focus on improving the results of contact tracing and completion rates of indicated LTBI treatment. Adherence is better with the short rifamycin-based regimens with no apparent difference in toxicity.

This study suggests that the role of the nurse case manager with TB expertise, telemonitoring and electronic review of the collection of medication from pharmacies may be key, especially in low-incidence countries, to improve preventive treatment adherence.

## Data availability statement

The raw data supporting the conclusions of this article will be made available by the authors, without undue reservation.

## Ethics statement

The studies involving humans were approved by Biomedical Research Ethics Committee of Euskadi PI2023097. The studies were conducted in accordance with the local legislation and institutional requirements. Written informed consent for participation was not required from the participants or the participants’ legal guardians/next of kin in accordance with the national legislation and institutional requirements.

## Author contributions

NO: Data curation, Investigation, Writing – original draft. IL: Conceptualization, Methodology, Writing – review & editing. JT: Data curation, Writing – review & editing. BT: Writing – review & editing. BS: Formal analysis, Writing – review & editing. LA: Data curation, Investigation, Writing – review & editing. JG: Data curation, Investigation, Writing – review & editing. ET: Methodology, Writing – original draft, Writing – review & editing, Investigation.
